# Experiences of Self-Criticism and Self-Compassion in People Diagnosed With Cancer: A Multimethod Qualitative Study

**DOI:** 10.3389/fpsyg.2021.737725

**Published:** 2021-10-13

**Authors:** Judith Austin, Constance H. C. Drossaert, Robbert Sanderman, Maya J. Schroevers, Ernst T. Bohlmeijer

**Affiliations:** ^1^Department of Psychology, Health and Technology, Faculty of Behavioural, Management and Social Sciences, University of Twente, Enschede, Netherlands; ^2^Department of Health Psychology, University Medical Center Groningen, Groningen, Netherlands

**Keywords:** self-criticism, self-compassion, cancer, qualitative research, multimethod

## Abstract

**Objective:** Self-criticism is a self-condemning and self-compassion a supportive style of self-to-self relating. These concepts have increasingly been studied in people with cancer, but mainly with quantitative studies. This study is the first to explore how adult cancer patients experience self-criticism and self-compassion in the context of their illness.

**Design:** A multimethod qualitative study design was used, combining individual and group semi-structured interviews. Participants were 26 people with cancer who familiarized themselves with the topic by doing various self-compassion exercises for 2 weeks prior to the interview. Individual and group interviews were analyzed together using thematic analysis.

**Results:** Four themes regarding self-criticism were identified: (1) being harsh or strict with yourself, (2) feeling guilty or angry, (3) feeling useless or like a burden, (4) feeling ashamed and not wanting to show weakness. Six themes regarding self-compassion were identified: (1) being mild to yourself, (2) guarding your boundaries, (3) accepting the illness and limitations, (4) maintaining a positive perspective, (5) connecting to others, and (6) taking responsibility for your health.

**Conclusion:** Our findings offer insights into practical and daily life experiences of self-criticism and self-compassion of people with cancer, which can aid the further development of theory, scales and interventions.

## Introduction

Living with cancer typically affects many aspects of a person’s life, including one’s physical, mental, and social well-being. Physical effects of the illness and treatment, such as fatigue, can impact a persons’ quality of life and diminish opportunities to participate in activities and roles that make life meaningful (Janda et al., 2000; Ann et al., 2015). Adjustment to cancer involves various processes of adaptation as one is managing, learning from and accommodating all the life changes incited by the diagnosis (Brennan, 2001). People with cancer are at an increased risk for experiencing mental distress, including anxiety and depression (Zabora et al., 2001; Trindade et al., 2018). An additional challenge in the adjustment process is that many people with cancer tend to be critical of their bodies, their lifestyle behaviors, their personality traits and how they are managing their illness and its consequences (Bennett et al., 2005; Przezdziecki et al., 2013; Callebaut et al., 2017).

Self-criticism can be described as a self-orientation that entails harsh self-scrutiny, negative self-evaluation and an excessive concern with personal failure (see Lueke and Skeel, 2017). Self-criticism can be a way to try to correct or improve the self, or to persecute the self for failures (Gilbert and Procter, 2006). This can present as explicit self-critical cognitions, but also as feelings of guilt, shame, or anger: these internalized defense strategies and resulting emotions often get fused (Gilbert and Procter, 2006). In clinical and non-clinical populations, self-criticism is associated with depressive symptoms (Warren et al., 2016), loneliness (Wiseman, 2005), and seeking less social support (Blalock et al., 1995).

Little is known about the experience and impact of self-criticism specifically in the context of cancer. It is assumed that in the face of a cancer diagnosis, people naturally try to make sense of the event by giving meaning to how and why it happened, or why the treatment is (not) progressing in a certain way. This process may involve guilt, shame, self-blame, and self-critical cognitions (Callebaut et al., 2017). One crossectional study found that high self-criticism is significantly associated with high distress in women with breast cancer (Campos et al., 2012). Furthermore, in a systematic review of self-blame in people with physical conditions, it was found that between 18 and 39% of people with cancer experience self-blame (Callebaut et al., 2017). Although self-criticism does not always concern causal attributions of the illness onset, it does seem to appear more in people with diagnoses strongly related to lifestyle-based risk factors (e.g., lung cancer) (Callebaut et al., 2017). On top of these self-condemning experiences, self-criticism often goes together with difficulties in generating reassuring and kind feelings toward oneself, i.e., difficulties in being self-compassionate (Gilbert, 2009).

In contrast to self-criticism, self-compassion can be described as an internal resource that enables responding to difficulties with kindness and wise, caring action. Self-compassion creates a context of relating to oneself that supports effective emotion regulation and social connection (Neff, 2013). While research on self-compassion in people with cancer is still in the early stages, more and more studies show that higher self-compassion in people with cancer is significantly associated with lower depression (Pinto-Gouveia et al., 2014; Todorov et al., 2019; Zhu et al., 2019), anxiety (Todorov et al., 2019; Zhu et al., 2019), and distress (Pinto-Gouveia et al., 2014; Todorov et al., 2019), lower body-image distress (Todorov et al., 2019), higher treatment adherence (Sirois and Hirsch, 2019), and higher resilience (Alizadeh et al., 2018). Evidence from intervention studies further demonstrates the relevance of self-compassion, as a recent review shows that training self-compassion skills via compassion-based interventions can yield reductions in anxiety, depression and self-criticism in people with cancer (Austin et al., 2020).

A limitation of existing research on self-compassion in cancer patients is the almost exclusive use of quantitative methods, e.g., correlational and intervention studies using self-report questionnaires to assess self-compassion. Although valuable in showing how self-compassion relates to patients’ outcomes such as distress and providing evidence for the efficacy of compassion-based interventions, these studies cannot shed led on cancer patients’ experiences of self-criticism and self-compassion. Having more insight into this may inform researchers and health care professionals whether and how compassion-based interventions should be adapted to the context of the cancer patient. In the aforementioned review by Austin et al. (2020), none of the compassion-based interventions were tailored to the distinct experiences of self-criticism and self-compassion or specific needs regarding compassion-based interventions of people with cancer. Only in one qualitative study by Lathren et al. (2018), experiences with an online Mindful Self-Compassion (MSC) group intervention of young adult cancer survivors were investigated. The participants expressed that training self-compassion helped them to address peer isolation, e.g., by becoming more mindful of the support already present in their environment and by learning to become a source of support for themselves. In addition, training self-compassion enabled the young adults to become kinder and more accepting instead of critical toward physical limitations and to experience gratitude for their working body parts. While these results indicate some interesting themes that are relevant for understanding experiences of self-compassion and benefits of compassion-based interventions in cancer patients, they do not address experiences of self-criticism and self-compassion of people with cancer of all ages and in various contexts. In general, in the field of self-compassion, there is a lack of qualitative research, with so far only two other qualitative studies on the experiences of self-criticism and self-compassion in adult clinical or non-clinical (non-cancer) populations (Pauley and McPherson, 2010; Halamová et al., 2018). These studies show that participants describe both emotional and action-oriented aspects of self-compassion. Participants describe self-compassion as meaningful, offering benefits such as an increased sense of calm, while also expressing unfamiliarity with the concept and perceived challenges such as high self-criticism (Pauley and McPherson, 2010; Halamová et al., 2018). Given the sparsity of qualitative studies, also in the context of cancer, there is a need for further qualitative research. The current qualitative study aims to explore in what ways people with cancer experience self-criticism and self-compassion, after prompting them with minimal and untailored self-criticism and self-compassion exercises to facilitate reflection on these often unfamiliar concepts. Such insights will increase our understanding of self-criticism and self-compassion in the process of adjusting to cancer and may inform future development and evaluation of theory, scales and (self-) compassion interventions.

## Materials and Methods

This study was approved by the Ethical Committee BMS of the University of Twente. A multimethod qualitative study design was used to study experiences of people with cancer regarding self-criticism and self-compassion. A semi-structured interview approach enabled an in-depth exploration of personal experiences and meaning (Carter and Henderson, 2005). Individual interviews were corroborated with group interviews in order to increase data richness. Specifically, group interviews can supplement the in-depth personal accounts of individual interviews with data emerging from group interactions about similarities and differences in experiences (Lambert and Loiselle, 2008). Individual and group interviews were conducted simultaneously and non-hierarchically, and thus were not informed by either outcome during the data collection phase.

### Participants and Procedure

Over the course of the year 2019, participants were recruited via convenience and snowball sampling, through the personal and social media networks of the interviewers and via oncology nurses at two hospitals. Prospective participants were contacted face-to-face, by telephone or by e-mail to provide them with information about the set-up of the study and to refer them to the study website. On the study website prospective participants could read information about the study (including data and privacy regulations), contact the researchers for questions and sign up for participation. For ethical considerations, participants were able to choose between participation in the individual or the group interviews, since contact with peers can be empowering but also emotionally challenging (Ussher et al., 2006). Group interviews were embedded within a series of co-design workshops (outside of the scope of this study). The prospective participant was called by the interviewer or researcher to discuss any further questions and to check the inclusion criteria.

The primary inclusion criterion was that participants received a diagnosis of cancer in the past 10 years. A sufficient command of the Dutch language and willingness to try out self-compassion exercises for 2 weeks prior to the interviews were also required (see below for explanation of the exercises). The study was part of an overarching research project which includes the development of a self-compassion intervention for people with cancer. Prospective participants were informed that their participation would help tailor this intervention and in turn help future patients. Indeed, the main reported reason for participation was to help future patients based on one’s own experience (in contrast, interest in self-compassion was rarely reported as a reason for participation).

After inclusion, the participant received a digital booklet with eight (self-)compassion exercises via e-mail. Since familiarity of participants with the concepts of self-criticism and self-compassion was expected to be low, these brief and untailored self-compassion exercises were offered in order to prompt reflection (see below for details about exercises). Participants were invited to try out the exercises during the 2 weeks prior to the interview, for 10 min a day. The individual interviews took place face-to-face (at the participants’ home or at the university) or by telephone, and lasted approximately 1 h. The individual interviewers were two master students in psychology with prior interviewing experience. The group interviews took place within a co-design workshop at the university or university medical hospital with 4–6 patients, 2–3 nurses, and 3 researchers with group-interview-experience present. The group interviews were conducted in 2 meetings of 1 h to account for the expected reduced energy level of people with cancer and since this was most fitting within the overarching co-design sessions. Thus, more time was allotted than for the individual interviews, to allow time for group interaction processes. Two groups were created based on geographical area, resulting in a total of 4 sessions of 1 h. During the group interviews, questions were first discussed in small groups of 2–3 patients, and were then discussed and summarized in the full group to allow for initial discussion of sensitive topics in a more intimate setting while also benefiting from the interaction of the larger group. Post-it notes and cards were used by participants to write down, visualize and sort their experiences, since such methods can facilitate idea generation (Diehl, 2009). For the group interviews, the first exercise and background information were also introduced during the meetings, while for the individual interviews exercises were exclusively completed at home. Written informed consent was obtained prior to all interviews. All sessions were audio-recorded and transcribed a verbatim.

### Materials

#### Exercises

Eight meditative and reflective exercises from existing (self-)compassion trainings MSC (Neff and Germer, 2018), Compassionate Mind Training (CMT) (Gilbert and Procter, 2006; Hulsbergen and Bohlmeijer, 2015; Irons and Beaumont, 2017; Van den Brink and Koster, 2019) were provided. The exercises were selected based on the aim of offering a variety of different types and modalities of exercises (e.g., reflection, visualization, audio-guided, writing). The exercises were accompanied with brief background information about self-criticism and self-compassion, and were not adapted to people with cancer. See Table 1 for an overview of exercises.

**TABLE 1:** Overview of the self-compassion exercises that participants tried out prior to the interviews.

Exercise	Type of exercise	Content of exercise	Source
Exercise 1: how do I treat a friend?	Reflective exercise	Reflect on possible differences between experienced self-compassion/criticism and compassion/criticism toward others	“How do I treat a friend?” from MSC
Exercise 2: three emotion systems	Reflective exercise	Reflect on emotional experiences by drawing Gilberts’ three emotion systems	“Three emotion systems” from CMT
Exercise 3: self-compassion mantra	Meditative exercise	Meditation in the form of a self-compassion mantra	“Self-compassion mantra” from MSC
Exercise 4: the flows of compassion	Reflective exercise	Reflect on the experience of giving compassion to yourself, to others, and receiving compassion from others	“The flows of compassion” from CMT
Exercise 5: metta/loving-kindness	Meditative exercise	Meditation in the form of a kindness exercise	MSC and CMT
Exercise 6: compassionate friend	Meditative exercise	Meditation that employs visualization to imagine a compassionate friend	MSC and CMT
Exercise 7/8: starting and closing the day	Reflective exercise	Starting with mindfulness and kind intentions, closing with reflecting on compassion	CMT (“Compassie als sleutel tot geluk”)

### Adherence and Acceptability of Exercises

Most participants reported that they engaged with the daily exercises entirely as intended by the researchers (77%). The remaining participants either did all exercises in 1 week instead of 2, did them in a different order, or skipped some exercises. Most participants spent about 5–15 min a day on the exercises, as intended. A few participants (15%) repeated the exercises a lot more often than intended and spent more time on them (e.g., repeating a one-time exercise every day). Most participants described that the exercises helped them to reflect on their situation (and specifically, their emotions and self-criticism) and to gain new insights, to take more rest, or to maintain a more positive perspective on life. Other participants wanted the exercises to be more practical and missed real-life (cancer-related) examples. While the reflective exercises appealed to all participants, experiences with meditative exercises were quite mixed (e.g., from very helpful to get rest and to be more kind, to very difficult to focus, difficult to visualize etc.).

### Interview Guide

The semi-structured interview guide consisted of three parts, and was devised to match the exercises. The first part was an introduction and included the aims, practical and background information of the study and the background and motivation of the interviewer. It also included questions about the diagnosis, treatment and well-being of the participant. The second part consisted of questions about the experiences of self-criticism, self-compassion and the exercises. After a few questions about adherence to and evaluation of the exercises in general, a set of questions was asked for each exercise. First, the participant was asked about the use, perceived effect and intention for further use of the exercise. Second, questions about the experience of self-criticism and (self-)compassion were asked that were related to the topic of the exercise. For example, for Exercise 1 “how do I treat a friend?” a question was “Do you recognize that you are more critical toward yourself than toward others? Can you give examples of in what ways you have been self-critical since the diagnosis?” And for Exercise 3 “self-compassion mantra” a question was “In a difficult moment, to what extent do you think you could remember to have compassion for yourself?” The questions related to the exercises also addressed compassion for others (outside the scope of this study). During the third part of the interview, participants were asked for demographic information (gender, age, marital status, employment status, education) and whether they knew any other potential participants. While the interview guide was identical for the individual and group interviews, for the group interviews the demographic- and illness related information was obtained in writing. See Appendix for the full interview guide.

### Data Analysis

A thematic analysis approach was used in order to minimize and describe the data (Braun and Clarke, 2014). Interview transcripts were first read by two researchers (JA, CD) to familiarize themselves with the data and to take notes of potentially interesting themes. Next, the two researchers selected text fragments that provided answers to the research questions (“In what ways are patients self-compassionate and self-critical?”). They then independently and inductively generalized initial codes, and subsequently sorted them into broader themes. The results were then discussed by the two researchers, and were reviewed at the level of the individual codes as well as the overall themes. Differences were solved by discussion, and this process was repeated iteratively until a final version of codes and themes was agreed upon. Results from the individual and the group interviews were analyzed together, while keeping track of the source of the findings to get an impression of similarities and differences. After inspecting the themes and codes, it was concluded that the minimal differences between group and individual interviews did not warrant separate analysis. Since themes from the group interviews emerged from interaction data (i.e., discussion between participants), citations are reported on a group level. All themes described in the results section are substantiated by data from both group and individual interviews (100%). The majority of underlying codes were substantiated by both group and individual interviews (66%).

## Results

### Study Sample

Participants were 16 females and 10 males with a mean age of 48 (range 22–78 years), of which 12 participated in the group interviews. The most common diagnoses were breast cancer (*n* = 10), leukemia (*n* = 2), colorectal cancer (*n* = 2), and testicular cancer (*n* = 2). Participants were diagnosed between 6 months and 10 years ago, with a mean of 3 years ago. The majority of participants had a theoretical education (81%), were married or in a relationship (77%), and were engaged in paid work or study (62%). Generally, participants were not familiar with the concept of self-compassion prior to this study.

### In What Ways Are Cancer Patients Self-Critical After Their Diagnosis?

**FIGURE 1:**
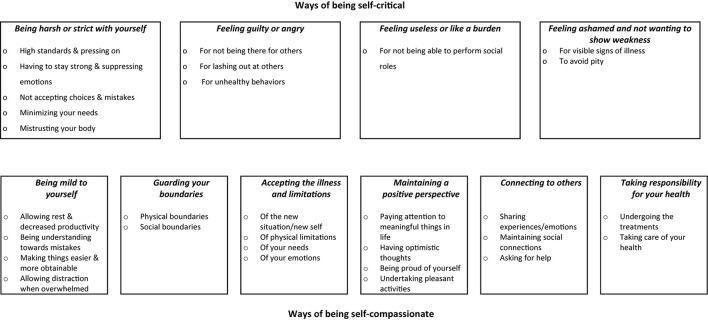
Ways in which people with cancer are self-critical and self-compassionate in relation to their diagnosis.

Participants described myriad ways of being self-critical after their diagnosis. This included illness-specific experiences (emotions/cognitions/behaviors) such as criticism about lifestyle and illness-management choices, not being able to fulfill previous social roles and tasks, and feeling shame for a changing physical appearance. See Figure 1 for a full overview of themes. While the illness seemed to illicit self-criticism for some, others described being less self-critical post-diagnosis, due to experiencing a “wake-up call” and a re-ordering of priorities (e.g., “I wonder what I was so critical of most of my life, like my weight…now I just think: you are who you are”^*I*^). Below we will explore the following ways of being self-critical in the context of a cancer diagnosis: being harsh or strict with yourself, feeling guilty or angry, feeling useless or like a burden, and feeling ashamed and not wanting to show weakness. (^*I*^ = quote from individual interview, ^*G*^ = quote from group interview).

### Being Harsh or Strict With Yourself

Many participants described being much more strict with themselves than with others. They pushed themselves to *set high standards and press on* [“Eating something unhealthy just for once would be okay, but I don’t allow it at all”^*I*^, and “sporting once (a week) is not okay, it has to be twice”^*I*^]; “If it happens to someone else, like a friend, then I would advise them: ‘just sit in that wheelchair, it’s okay.’ But for yourself you’re just much harsher, that’s the difference”^*I*^). Participants described *having to stay strong and suppressing emotions*, for example by not asking for help: “I already had symptoms for a long time, and still I did not go to the hospital”^*G*^), and “you’re sad, but then you tell yourself instantly: come on, get your act together, you have to go on!”^*G*^). *Not accepting choices and mistakes* (“I just need to do things right. If someone else makes a mistake that’s not as bad as when I make a mistake”^*I*^), *minimizing your needs* (“I did not want to bother others, they are probably not looking for an afternoon of crying”^*G*^, “I was quick to think: come on, it’s not that bad”^*I*^) and *mistrusting your body* (“the trust in your own body, it is just gone”^*I*^) were further ways of being harsh and strict with yourself.

### Feeling Guilty or Angry

Many participants reported feeling guilty or angry toward themselves. For example because of *not being there for others*, both emotionally (“she was struggling a lot, and I did not notice it, I was just focused on myself”^*G*^, and: “I could not listen to other people’s problems anymore…you hear that someone has sore feet, and you think: ‘who cares”’^*G*^) and physically (“I had to cancel again”^*I*^, “we share a business together, and that is hard for him, he has to work more now”^*G*^). Others described feeling angry or guilty because of *lashing out at others* (“I lose my patience much quicker, and then I am angry at myself later”^*G*^), and “I don’t manage to be friendly all the time, especially when I’m tired”^*G*^). Participants also felt guilty because of self-proclaimed *unhealthy behaviors* (“I could not get myself off the couch…and then I felt lazy, like the situation is my own fault”^*I*,^ and “if I order a pizza and watch Netflix, I feel really bad. Then I think, everyone, the doctors and the nurses, they all recommend a balanced diet and a lot of exercise”^*G*^). Another example was not consulting a doctor earlier (“could I have gone earlier, could I have discovered something earlier?”^*G*^).

### Feeling Useless or Like a Burden

Symptoms of the illness and side-effects of the treatment frequently made participants *unable to perform social roles*. This often elicited an experience of feeling useless: “When one of your children is moving, you cannot help. I could join for a little bit, but to say that I was of any added value…no”^*G*^, and “where did that time go, when they asked you to help with something?”^*G*^). Participants further described “feeling useless because you cannot work anymore”^*G*^ and for not contributing to society: “everything has stagnated…I can only undergo treatments…I am just outside of society”^*I*^). Some participants reported feeling like a burden to others: “you don’t want to be a burden…everyone is already busy with all sorts of things, and then you come and add your shit”^*G*^).

### Feeling Ashamed and Not Wanting to Show Weakness

Participants were confronted with feelings of shame for *visible signs of the illness*: “I had a lot of fluid retention, and that made me so insecure”^*I*^ and “when I walked pass a mirror I did not like it at all, I just didn’t find short hair beautiful”^*I*^. This also relates to *avoiding pity* from others: “Your illness becomes very visible…when you are bald you obviously have cancer. Then you are clearly doing terribly, you are pathetic”^*I*^.

### In What Ways Are Cancer Patients Self-Compassionate After Their Diagnosis?

Participants described many ways of being self-compassionate after their diagnosis. This included being mild to yourself and lowering self-demands, intentional self-care behaviors such as taking care of your health and undertaking pleasurable activities, as well as a more general surrender to the new situation by accepting accompanying limitations. See Figure 1 for a full overview of themes. Next to the previously mentioned ways of being self-critical, many participants described being more self-compassionate post- diagnosis, for instance because the suffering they encountered left them no choice but to prioritize their own health and well-being (e.g., “I never needed self-compassion as much as I do now”^*I*^). Below we will explore ways of being self-compassionate in relation to the cancer diagnosis: being mild to yourself, guarding your boundaries, accepting the illness and limitations, maintaining a positive perspective, connecting to others and taking responsibility for your health.

### Being Mild to Yourself

Participants reported a variety of ways of being mild, for example by *allowing rest and decreasing self-demands for productivity* (“I am allowed to take time to rest. I am allowed to feel really tired…and I am allowed to just lie down on the couch”^*I*^) and “you don’t have to do it all…I cannot live my life the way I did before”^*I*^, and “the nurse advised me to go and take a walk around the estate…and I thought: for me the kitchen is far enough”^*I*^). Other ways of being mild are to *be understanding toward yourself when you make mistakes* (“I know I was wrong and I admitted that, but I don’t have to persecute myself later just because I had a human emotion”^*I*^) and *making things easier or more obtainable for yourself*: “the treatment left me with very dry skin and I have to apply body lotion every day… now I put on some music to make it less of an obligation and more pleasant”^*I*^). *Allowing distraction when things get too overwhelming* (“just focus on other things for a while…for me my infant son helped a lot with that”^*G*^) also was a way of being mild for yourself.

### Guarding Boundaries

Guarding boundaries was important to most participants, both regarding social contacts and regarding physical activity. *Social boundaries* included communicating toward others that you are not feeling well and cannot participate (“When I really can’t take it anymore, I can now communicate clearly: I am going to lie in bed now”^*I*^). It also regards making choices about which contacts to invest energy in and which not (“I said: ‘I prefer that you don’t ask me how I’m doing…I will send you an update myself,’ because otherwise it was just too much”^*I*^, and “my whole life I adjusted to her, but when I got the diagnosis I said: ‘now it is done,’ you have to adjust now.”^*G*^). *Physical boundaries* were set by listening to your body (“I took the time to listen to my body…at first I said that I would continue working but then I noticed…no, I think it’s wise if I go home early”^*I*^) and by alternating rest with activity (“if there is a birthday to go to in the evening, then I don’t do anything during the day, and I don’t do anything the day after either”^*G*^).

### Accepting the Illness and Limitations

Participants described accepting *the situation and their “new self”* [“I was able to accept: ‘okay,’ it is what it is…this is what happened”^*I*^, and “it’s okay to be a different (first name) now, after everything I have been through”^*G*^] as a way of being self-compassionate. Furthermore, *accepting physical limitations* (“I thought I have to do this and this and…. but I can’t do it all, and that’s just how it is”^*G*^), *accepting your own needs* (“when I heard I had cancer, I thought: ‘now I will think of my own needs again”’^*I*^) and *accepting and giving space to your emotions* (“if there is sadness or pain, you don’t have to ignore it…you can give yourself the chance to dive in for a while”^*I*^) were described.

### Maintaining a Positive Perspective

Another way to take care of yourself in difficult times is *paying attention to meaningful things in life* (“I feel like I live more intensely, that I really enjoy things…like watching a little bird in a tree”^*G*^, and “I celebrated my birthday, despite everything”^*I*^). *Having optimistic thoughts* (“your chances to recover are good, that brings positivity…and if nothing can be done then you just have to make the best of the time that’s left”^*I*^ and “when you are having a hard time, you remind yourself that that is part of life”^*I*^) was important, also in order to encourage yourself: “I was continuously encouraging myself in my thoughts”^*I*^. Participants described *being proud of themselves* regarding what they were still able to do: “before, I could do this for 3 h, now only 1….then I realized: I am already doing great”^*G*^. *Undertaking pleasant activities* was another way for participants to feel positivity (“sometimes I go to bed nice and early with a cup of tea and a book, and I think: I am going to take care of myself now, let the word turn without me”^*I*^, and “doing nice things with a friend”^*G*^).

### Connecting to Others

Participants described taking care of themselves by connecting to others, for example by *sharing experiences and emotions* (“we really take the time to talk about it, because for now this is the most important thing that’s happening”^*I*^, and “I talk about it a lot…it helps when you can tell your story…after telling it 5 times you have a different perspective than when you told it the first time”^*I*^). Others described *maintaining social connections*, even when you cannot be around as much (“keep in touch with work…that brought me a lot, to just keep in touch with colleagues”^*G*^). *Asking for help* was also important (“I noticed that I can really communicate my needs well when I need help”^*I*^, and “look for other patients who know what you’ve been through”^*G*^).

### Taking Responsibility for Your Health

*Undergoing the treatments* was described as a way to take care of yourself (“the time you invest every week to go to the hospital, for your own health…to just allow that and realize that it’s okay”^*I*^, and “I tried to get through the chemo by just lying there and relaxing”^*I*^). *Taking care of your health* was also important [“I was not supposed to eat red meat anymore, and I used to love it…but I don’t do it because I want to stay healthy, that’s a way in which I am kind to myself”^*I*^ and “I was trying to get in shape in between treatments (with exercise), and with each treatment what I built up was broken down, but it helped me a lot”^*G*^].

## Discussion

This is the first qualitative study that investigated ways in which adults with cancer are self-critical and self-compassionate in relation to their diagnosis and illness. All participants were able to describe ways of being self-critical as well as being self-compassionate post-diagnosis, often reporting being self-critical in some areas (e.g., about lashing out at others) and being self-compassionate in other areas (e.g., by taking rest).

Four themes describing ways of being self-critical were identified: being harsh or strict with yourself, feeling guilty or angry, feeling useless or like a burden, feeling ashamed and not wanting to show weakness. Being harsh or strict with yourself is a theme that has been identified by Neff (2013) as the opposite of self-kindness and is also a facet assessed by the Self-Compassion Scale (Neff, 2016). However, the other themes brought up by patients have not gotten much attention in existing self-compassion scales and literature. Interestingly, when looking beyond the literature on self-compassion and expanding to literature on coping with cancer, some resemblance with our results can be noted, particularly regarding the experience of feeling useless or like a burden (Refsgaard and Frederiksen, 2013). It is possible that this entails a form of self-criticism more specific to people with cancer or perhaps long-term physical conditions in general, brought on by an inability to perform various social roles and by being dependent upon others. Another way in which self-criticism may express itself in people with long-term physical conditions, is that stigma regarding diagnoses strongly related to lifestyle-based risk factors (e.g., lung cancer) may become internalized by patients in the form of harshness or guilt, even when they did not engage in these harmful lifestyle behaviors themselves (Else-Quest et al., 2009; Marlow et al., 2010). Further research is warranted to investigate the experience of these types of self-criticism in people with cancer and other long-term physical conditions, and the extent to which these experiences are similar to or different from other populations.

Six themes describing ways to be self-compassionate were identified: being mild to yourself, guarding your boundaries, accepting the illness and limitations, maintaining a positive perspective, connecting to others and taking responsibility for your health. Self-compassion as experienced by people with cancer thus encompasses coming to terms with the situation, resting, setting boundaries and lowering expectations (“doing less”), while also taking active steps to take care of oneself by focusing on positive aspects of life, sharing with others and engaging in healthy behaviors (“acting more”). Similarly, theoretical definitions of self-compassion describe dimensions of noticing and being with suffering, as well as acting to alleviate suffering (Gilbert, 2014; Neff and Germer, 2018) as corroborated by the few qualitative studies of self-compassion in other adult populations (Pauley and McPherson, 2010; Halamová et al., 2018). Thus, although self-compassion is sometimes publicly (mis)understood as overly soft or accepting (Gilbert et al., 2019), our results concur that self-compassion requires courage and wisdom to make beneficial, albeit difficult, decisions. In that way self-compassion contributes both to recovery (e.g., by taking rest) and sustainability and growth (e.g., by redefining priorities post-diagnosis) aspects of resilience in the adaptation process (Austin et al., in press).

Some elements of theoretical approaches to self-compassion can be recognized in our results. For example, being mind to yourself resembles an aspect of self-compassion identified by Neff (2013) as self-kindness. However, our other themes are not measured by Neff’s Self-Compassion Scale (SCS), and these have mainly been overlooked by previous quantitative research on self-compassion. We suggest that this is because our findings particularly shed light on a more practical, daily life experience of self-compassion (e.g., specific emotions, behaviors and cognitions)—something that earlier (more conceptual) qualitative studies of self-compassion in adult populations have also not addressed (Pauley and McPherson, 2010; Halamová et al., 2018). Our findings thereby help to bridge the gap between theoretical constructs and lived experience. This connection may be useful in the development or adaptation of compassion-based interventions for people with cancer, making the concept of self-compassion more relatable to this target population and hopefully increasing intervention success. Indeed, in our own development of a mobile self-compassion self-help training for people with cancer (Austin et al., 2020) the themes found in this study are embedded as topics to be addressed in the training. For example, “taking responsibility for your health” as an intervention topic addresses the difference between self-compassion and self-indulgence (e.g., undergoing treatments that may be unpleasant at first but that reduce suffering in the long-term), as well as practicing compassionate self-correction and goal setting (e.g., practicing understanding when a health behavior goal is not met, and then supporting yourself by adjusting the goal or facilitating circumstances that make it more obtainable). While initial responses to the intervention are positive, only future research can tell how this applied approach to self-compassion will be perceived. Furthermore, our findings raise an interesting question about whether available instruments that measure (trait) self-compassion (e.g., the Self-Compassion Scale, Neff, 2016) align with the more practical descriptions of self-compassion found in this study, or whether more practical, tailored instruments could be of added predictive value. For example, the themes of the current study could translate to cancer-specific questionnaire items (e.g., an item for “guarding your boundaries” could be: “When coping with the limitations resulting from my illness, I indicated my boundaries to others.,” and an item for “feeling useless or like a burden” could be: “In relation to my illness, I feel guilty about being a burden to others”).

Research shows that self-compassion is inversely associated with self-criticism (Zhang et al., 2019). As Neff (2013) posits, self-criticism asks whether you are good enough, while self-compassion asks what is good for you. In our results, self-criticism and self-compassion sometimes presented as opposite responses, where for example self-criticism entailed not accepting your mistakes, and self-compassion entailed being understanding toward your mistakes. At other times this dynamic was more complex, for example when self-compassion was experienced as setting social boundaries and not being available to others, while self-criticism was experienced as feeling guilty for not being available to others. Indeed, what is experienced as self-compassion or self-criticism depends on what is needed to alleviate suffering in any given situation. Self-compassion can be seen as an alternative to self-criticism, wherein compassionate self-correction instead of self-criticism is practiced in response to failures or difficulties (Gilbert, 2010; Hulsbergen and Bohlmeijer, 2015). However, more research is needed to clarify how self-criticism and self-compassion relate, i.e., as two unipolar constructs or as opposite ends of the same spectrum. Intervention research indicates that when self-compassion is trained, a self-compassionate response becomes more available and a self-critical response may become less appropriate (Gilbert, 2010). Most compassion-based interventions target both self-criticism and self-compassion, and experienced benefits of less isolation/more connection and more acceptance toward physical limitations have been reported post-intervention (Lathren et al., 2018; Austin et al., 2021). Noteworthy, in our study the difference between self-criticism and compassionate self-correction was not always clear to participants, with self-criticism occasionally being described as something valuable and constructive. At times it was challenging to determine whether this indicated a meaningful difference in self-to-self relating or merely a difference in semantics. After the initial interviews, we began to clarify this by asking (more) follow-up questions regarding intentions preceding the “self-criticism” and feelings resulting from the “self-criticism.” We suggest that this is something to take into account and allot sufficient time for in future research of perceived self-criticism and self-compassion.

This study was strengthened by the integration of two different qualitative methods, which allowed for a more comprehensive investigation of experienced self-criticism and self-compassion in people with cancer. Similar to the study by Lambert and Loiselle (2008), we noticed that an individual interview typically offered a more linear perspective of antecedents and context of self-critical or self-compassionate experiences, while in group discussions participants were triggered by each other’s experience to remember similar examples, offering less detail but more spontaneity and breadth—despite the similarity in topics. The integration of different qualitative methods can indeed offer a more comprehensive view of complex experiences (Frank et al., 2019), although pitfalls such as assigning hierarchy to findings from different methods should be avoided (Lambert and Loiselle, 2008). Another strength of this study was that the use of self-compassion exercises prior to the interviews enabled participants to reflect on their experiences of self-criticism and self-compassion in the context of their daily lives. The exercises were kept brief and untailored for this purpose, which facilitated prompting experiences without a main focus on an intervention process. Given that most participants were not yet familiar with self-compassion, we believe that this was successful in increasing data richness. Nevertheless, it should be noted that this approach may have steered the results to some extent, since we did not capture the perspective of a “naïve” patient that a health care professional would most likely encounter in clinical practice. Conducting interviews prior to and after engaging with exercises could shed more light on this perspective. Noteworthy, a limitation of this study is that 5 out of the 26 participants were diagnosed a long time ago (8–10 years), and their experiences of self-criticism and self-compassion in relation to their diagnosis and illness may be clouded by other, more recent, life experiences. Furthermore, while compared to other studies our sample was quite balanced in terms of gender, the majority of participants had a theoretical education, were married/in a relationship and were from a non-migration background. In addition, breast cancer was by far the most common diagnosis. Therefore it is unclear whether our findings apply to other groups of cancer patients, such as people with a vocational education, for whom rates of self-compassion have been found to be lower than for people with a theoretical education (López et al., 2018).

In sum, our multimethod qualitative study described experiences of self-criticism and self-compassion in people with cancer in relation to their diagnosis and illness. Four themes describing ways of being self-critical were identified: being harsh or strict with yourself, feeling guilty or angry, feeling useless or like a burden, feeling ashamed and not wanting to show weakness. While existing research focuses more on experienced guilt, shame and anger, being harsh or strict with yourself and feeling useless or like a burden warrant further attention. More research is needed to investigate these and other specific ways in which people with cancer are self-critical, and how they are similar to or different from other (illness) populations. Six themes describing ways to be self-compassionate were identified: being mild to yourself, guarding your boundaries, accepting the illness and limitations, maintaining a positive perspective, connecting to others and taking responsibility for your health. Elements of theoretical approaches to self-compassion can be recognized in our results, including dimensions of “being with” as well as “acting to alleviate” suffering. However, our findings particularly offer insight into the practical and daily life experiences of self-compassion, with may prove to be helpful in the further development of theory, scales and interventions.

## Data Availability Statement

The datasets presented in this article are not readily available because of the sensitive nature of this research (interview transcripts are not available for sharing). Requests to access the datasets should be directed to corresponding author.

## Ethics Statement

The studies involving human participants were reviewed and approved by the Ethical Committee BMS of the University of Twente. The patients/participants provided their written informed consent to participate in this study.

## Author Contributions

JA and CD performed the material preparation, supervision of individual interviewers, and data analysis. JA wrote the first draft of the manuscript. All authors commented on previous versions of the manuscript, read and approved the final manuscript, and contributed to the study conception and design.

## Conflict of Interest

The authors declare that the research was conducted in the absence of any commercial or financial relationships that could be construed as a potential conflict of interest.

## Publisher’s Note

All claims expressed in this article are solely those of the authors and do not necessarily represent those of their affiliated organizations, or those of the publisher, the editors and the reviewers. Any product that may be evaluated in this article, or claim that may be made by its manufacturer, is not guaranteed or endorsed by the publisher.

## Appendix

### Interview Guide

Part I: diagnosis, treatment and well-being of participant

• Introduction: aims, practical and background information of the study and the background and motivation of the interviewer.
• Illness-information: diagnosis, time since diagnosis, (experience of) received treatment, how the participant currently is doing.

Part II: experiences of self-criticism, self-compassion and the exercises

Brief evaluation of exercises (use, appreciation, perceived effect and intention for further use) in general and per exercise.

1. What is your impression of the concept self-compassion(/self-criticism)? Why does or doesn’t self-compassion(/self-criticism) fit with the situation of people with cancer?

[Exercise 1]

2. Can you think of examples when you were self-critical? Can you think of examples when you were self-compassionate?

[Exercise 2]

3. In what ways do you recognize the three emotion systems in your own life? Has your cancer diagnosis changed anything in the balance between the three systems?

[Exercise 3 and 6]

4. How would you describe self-compassion in your own words? (examples) Is this different after the diagnosis compared to before?
5. In a difficult moment, to what extent do you think you could remember to have compassion for yourself? To what extent are you able to have distance from a difficult situation, or are you overwhelmed by thoughts and feelings? How was this in the time after the diagnosis?
6. Did the diagnosis affect how connected or isolated you feel from other people? (examples)

Note: Additional questions were asked about giving and receiving compassion [Exercises 4 and 5], which were not part of the current study. There were no specific questions for [Exercise 7/8]; this reflection exercise already included questions such as “In what ways was I kind to myself today?” which were during the evaluation of the exercise.
Part III: demographic information and wrapping up

• Are there any topics that we have missed? Is there anything that you would like to add?
• Demographic questions (gender, age, marital status, employment status, education).
• Wrapping up: asking if the participant knows any other potential participants, asking whether participant wants to receive study results.
